# Clinical Lectures upon Surgical Cases in the Buffalo Hospital of the Sisters of Charity

**Published:** 1870-10

**Authors:** J. F. Miner


ART. IV.—*Clinical Lectures upon Surgical Cases in the Buffalo Hospital of the Sisters of Charity* By. Prof. J. F. MINER, M. D.

REPORTED BY W. W. MINER, A. B., MEMBER OF THE CLASS.

---

GENTLEMEN —In inviting your attention to the cases which may present themselves at the Hospital, it may be well to remind you of a truth as yet but partially appreciated;—that Clinical observation is essential to surgical education. Whatever may be your theoretical knowledge you cannot be regarded as surgeons until you have had also some opportunities of practical observation. For the present, your stock of practical knowledge must depend upon such opportunities as are afforded in the College course. With this view every every effort has been made to make this source of instruction ample and satisfactory; both of our Hospitals are open to you, and many cases of disease, as well as most of the operations made by surgeons, will be presented and the operations made in your presence. It is greatly to be desired that you loose none of these opportunities of observation, since this will constitute for the present an important part of your available surgical knowledge. The minor operations, and the less important cases in one sense, claim your closest attention, since it is to this class that you are to mainly devote your attention and obtain your surgical standing. It is not in operations for stone, or tumors, or aneurism, that you are to commence your surgical reputation; but rather in your ministrations to the lesser ills which maim mankind. Hoping that you will appreciate the importance of the most trivial cases, as well as the attractions of the major operations, and having thus briefly introduced the general subject, permit me to invite your attention to

CASE I.—*Pterygium.*—P—— D—— has upon the inner angle of the eye a small, elevated growth, which is seated in, or just beneath, the conjuctival membrane, and whose border is of triangular outline. Severe pain exists in the eye at times, and the apex of the growth is gradually advancing upon the cornea, so that if it is allowed uninterrupted progress it will eventually interfere with vision. Closer examination shows the growth to be thickly traversed by a net-work of blood vessels, giving the whole a reddish tinge. The case presents the characteristic features of pterygium as to its vascularity, triangular form and location in the inner quadrant of the eye. The only way to get rid of pterygium is by means of an operation for which there are different methods. In the present case, the body of the growth is seized and hooked up by the forceps, while by means of a curved scissors the forward part is dissected up from the cornea and the remaining portion divided close down upon the sclerotic coat. The wound is not of large extent, and the eye will be dressed with a simple wet compress.

CASE II.—*Granular Lids.*—As I evert the upper lid you can see the reddened appearance of its lining membrane. Observing it closely you can see that it is studded with red elevations, which are very sharp and pointed. These granulations roughen the naturally smooth surface of the lid, and every time the lid is closed, or the globe moved, friction is made upon the delicate membrane of the cornea; it is thus rendered vascular and opaque. Almost always, when this disease has continued for any length of time, we have first, the granular lids; and then, as you see in this case, the vascular and opaque cornea. The condition of the cornea is the source of danger, and to it you are to direct attention. I say, direct attention, but not treatment. To judge of the severity of disease and the probable result, you are to observe the cornea; if any dangers to vision are present they are to be found in the condition of the cornea, and not to any changes which may have taken place in the lining membranes of the lids. A great variety of collyria have been recommended, and many plans of procedure proposed, but modern experience justifies the conclusion that a crystal of sulphate of Copper applied to the granulations every day, or every other day, in the manner that you now observe me make the application—by everting the lids and drawing it smoothly and lightly over the surface, is the most satisfactory and successful method of treatment. Nothing but want of perseverance and lack of thorough application can cause failure. You may, in such cases, apply it and nothing else; never being discouraged or deviating for a better remedy, for if properly applied it will certainly effect a satisfactory result. It is a safe, certain, and satisfactory remedy.

Case III.—*Amputation of Foot.*—The patient had his feet frozen some thirty years since, and has lost the phalanigeal extremities of both feet. There is now an ulcer seated in the cicatrix on the right foot, which gives the man great pain and uneasiness, and seriously interferes with the use of the limb. The vessels of the leg are varicose, which shows that the general circulation of the extremity is somewhat impaired, while circulation in the cicatricial tissue—which is the seat of the ulcer—is, of course, not so vigorous as would be in normal parts. Stimulating applications are often made in such cases—probably have been in this; but the idea at present is to obtain healthy flaps in normal tissue and secure, if possible, their union by first intention. Hey's operation by separating the tarso-metatarsal articulation, is found to leave too much bone for the flaps which the foot affords; therefore, the first and third cuneiform bones are sawed off even with the second cuneiform and the cuboid, thus giving an opportunity to nicely approximate the edges of the flaps. At a period two weeks after, the object of the operation appears to have been attained.

Case IV.—*Injury of the Eye.*—A boy, aged ten years, has lost the integrity of his left eye from having had brick-layers' mortar thrown into it some three months since. The cornea may have been pierced at the time of the injury, or an ulcer of the cornea may have resulted in the perforation which is now apparent, and through which the iris is seen protruding, forming what is improperly called hernia of the iris. Vision in the eye is altogether lost, and in order to promote the healing of the parts the protruded portion of the iris is snipped off and removed with the scissors, and the eye dressed with a light compress.

